# Impact of swapping soils on the endophytic bacterial communities of pre-domesticated, ancient and modern maize

**DOI:** 10.1186/s12870-014-0233-3

**Published:** 2014-09-12

**Authors:** David Johnston-Monje, Walaa Kamel Mousa, George Lazarovits, Manish N Raizada

**Affiliations:** Department of Plant Agriculture, University of Guelph, 50 Stone Road, Guelph, ON N1G 2W1 Canada; A&L Biologicals, Agroecology Research Services Centre, 2136 Jetstream Road, London, ON N5V 3P5 Canada; Department of Pharmacognosy, Mansoura University, Mansoura, 35516 Egypt

**Keywords:** Endophyte, *Zea*, Maize, Bacteria, 16S, Domestication, Evolution, Microbial ecology, Root, Shoot, Seed, TRFLP, Soil, Teosinte, Parviglumis, Mixteco, Landrace, Vertical transmission, Yield stability, Corn hybrid, Maize hybrid, Breeding

## Abstract

**Background:**

Endophytes are microbes that live within plants such as maize (corn, *Zea mays* L.) without causing disease. It is generally assumed that most endophytes originate from soil. If this is true, then as humans collected, domesticated, bred and migrated maize globally from its native Mexico, they moved the species away from its native population of endophyte donors. The migration of maize persists today, as breeders collect wild and exotic seed (as sources of diverse alleles) from sites of high genetic diversity in Mexico for breeding programs on distant soils. When transported to new lands, it is unclear whether maize permits only selective colonization of microbes from the Mexican soils on which it co-evolved, tolerates shifts in soil-derived endophytes, or prevents colonization of soil-based microbes in favour of seed-transmitted microbes. To test these hypotheses, non-sterilized seeds of three types of maize (pre-domesticated-Mexican, ancient-Mexican, modern-temperate) were planted side-by-side on indigenous Mexican soil, Canadian temperate soil or sterilized sand. The impact of these soil swaps on founder bacterial endophyte communities was tested using 16S-rDNA profiling, culturing and microbial trait phenotyping.

**Results:**

Multivariate analysis showed that bacterial 16S-rDNA TRFLP profiles from young, surface-sterilized maize plants were more similar when the same host genotype was grown on the different soils than when different maize genotypes were grown on the same soil. There appeared to be two reasons for this result. First, the largest fraction of bacterial 16S-signals from soil-grown plants was shared with parental seeds and/or plants grown on sterilized sand, suggesting significant inheritance of candidate endophytes. The *in vitro* activities of soil-derived candidate endophytes could be provided by bacteria that were isolated from sterile sand grown plants. Second, many non-inherited 16S-signals from sibling plants grown on geographically-distant soils were shared with one another, suggesting maize can select microbes with similar TRFLP peak sizes from diverse soils. Wild, pre-domesticated maize did not possess more unique 16S-signals when grown on its native Mexican soil than on Canadian soil, pointing against long-term co-evolutionary selection. The modern hybrid did not reject more soil-derived 16S-signals than did ancestral maize, pointing against such rejection as a mechanism that contributes to yield stability across environments. A minor fraction of 16S-signals was uniquely associated with any one soil.

**Conclusion:**

Within the limits of TRFLP profiling, the candidate bacterial endophyte populations of pre-domesticated, ancient and modern maize are partially buffered against the effects of geographic migration --- from a Mexican soil associated with ancestral maize, to a Canadian soil associated with modern hybrid agriculture. These results have implications for understanding the effects of domestication, migration, *ex situ* seed conservation and modern breeding, on the microbiome of one of the world’s most important food crops.

**Electronic supplementary material:**

The online version of this article (doi:10.1186/s12870-014-0233-3) contains supplementary material, which is available to authorized users.

## Background

Microbial endophytes live non-pathogenically inside their host plants and can provide a number of beneficial functions for their hosts, including aiding with nutrient acquisition, producing stimulatory plant hormones and antagonizing pathogens [1,2]. Endophytes benefit from living inside plants by gaining access to nutrients and protection from outside competition and predation [3]. As described below, there are conflicting reports concerning the immediate sources of endophytes, and the extent to which they are taken up from the surrounding environment (primarily soil) or inherited (vertically transmitted) [4]. A critical stage for soil microbes to gain access to plants would be during germination and early development, to become founders of the endophytic microbiome of adult plants.

Soil is considered to be the major environmental source of plant associated bacteria [5-9], and it is thus not surprising that roots are reported to be the most heavily colonized plant organ [10]. Textbook examples of soil derived microbes inhabiting plants include vesicular arbuscular mycorrhizae [11] and nodule-forming, nitrogen-fixing rhizobia [12]. Because they are not inherited through seed, rhizobia must re-infect legume roots every generation [13]. As such, when the legume soybean was introduced into the Americas, far away from its native Asian soil [14], its yields were low due to a lack of compatible soil rhizobia in the New World. To fix this problem, crude soil field transplants, and later inoculation with strains of pure soil inoculants of rhizobia, were used [15-18]. It remains to be established whether non-legume crops such as maize can benefit from soil microbes (as endophyte partners) that are located at their ancient sites of domestication.

Contrary to an environmental origin, there is evidence that in some plant species, bacterial endophytes can be inherited from one generation to the next through seed [19-29]. This behaviour would obviously be most advantageous for microbes that are the first to colonize a seedling, ensuring effective colonization of the new niche.

Understanding whether endophytes in young plants are primarily inherited or selected from a local soil has relevance to modern agriculture. Today, crop genotypes are shifted around the world and grown on new soils to facilitate breeding or *ex situ* conservation in seed banks where the seeds are re-grown periodically on foreign soil to maintain viability. Soil is considered to be the most microbially diverse habitat on Earth [30]; in fact, geographically distant soils within the Americas share only 4% similarity at the operational taxonomic unit (OTU) level [31]. If crops use soils as a passive “marketplace” for endophytes [8], then their associated bacterial communities are being significantly altered from soil to soil with unknown impacts.

*Zea mays* spp. *mays* (maize/corn) is one of the world’s three most important food crops. It is an example of a cultigen in which wild, exotic and modern genotypes are shifted around the world to facilitate breeding programs and *ex situ* conservation [32]. Maize is believed to have been domesticated in southern Mexico about 9,000 years ago in the state of Oaxaca from a wild grass ancestor whose closest living relative today is the wild teosinte, *Zea mays* spp. *parviglumis* (Parviglumis) [33]. The only significant natural population of Parviglumis that remains today is in the Balsas River valley of Mexico [34]. Following domestication, pre-Columbian farmers selected maize landraces to suit local environments and needs [35]. Christopher Columbus noted arriving in the Americas to see maize landraces being grown in massive fields 30 km long [36]. One of the most ancient surviving landraces, a giant plant called Mixteco (*Zea mays* ssp. *mays,* var. Mixteco), is still grown by Mexican farmers on acidic, nutrient poor soils and may represent a “missing link” between wild teosinte and modern maize [35]. In contrast to geographically adapted landraces, modern maize hybrids are the result of commercial breeding programs where the goal is to have stable yields across a diversity of soil types and environments [37]. Most of this breeding is now performed by companies, under high input conditions (e.g. fertilizers), rather than by local farmers, with as much as 94% of breeding in the United States conducted by the private sector [38]. Pioneer 3751 (*Z. mays* ssp. *mays*, Pioneer hybrid 3751) is an example of a modern maize hybrid that is grown on diverse temperate soils around the world including Canada, the United States and Europe. Pioneer 3751, grown on an agricultural soil in Wisconsin (USA), has been shown to contain at least 74 different phylotypes of bacteria within its roots [39].

As the center of origin, Mexico boasts the greatest genetic diversity of the above ancestral, exotic and modern maize [33]. These seeds are housed in a vault at the International Maize and Wheat Improvement Center (CIMMYT) in Mexico. From here, seeds are shipped to many other nations to facilitate breeding, but the impact of this seed movement on maize endophyte community composition has not been well characterized. Some evidence suggests that maize can take up endophytes from the soils it is adapted to grow on, and hence would be affected by migration: for example, an endophytic strain of nitrogen-fixing *Burkholderia* could only be isolated from a Mexican maize landrace when it was inoculated with its native agricultural soil [40]. In contrast, a previous study conducted by us showed that the relative bacterial endophyte composition of seeds from diverse *Zea* genotypes, imported into Canada from other nations, including Mexico, remained largely conserved when the plants were subsequently re-grown and seed harvested on Canadian soil [41]. This result suggested that *Z. mays* plants used vertical transmission of microbes to buffer their endophytic communities against geographic migration.

Several hypotheses might be expected to predict the effects of geographic migration on the endophyte populations of *Zea mays*. Like a sponge, *Z. mays* plants might passively acquire the majority of their bacterial endophytes from soil, resulting in dramatic shifts to endophyte populations when plants are grown on geographically distinct soils. It is also possible that *Z. mays* plants are able to discriminate between soil microbes, allowing only selective entry. It is tempting to speculate that pre-domesticated and other wild relatives of maize are genetically programmed to selectively uptake specific microbes that are only present in the soils on which these plants evolved [42]. In parallel, perhaps recent crop breeding for improved yield stability across diverse geographic locations has caused modern *Z. mays* to restrict entry or survival of microbes from diverse soils. Another possibility is that *Z. mays* inherits most of its microbiome through seeds rather than from the soil, buffering the plant’s endophytic communities against the effects of geographic migration.

**Figure 1:**
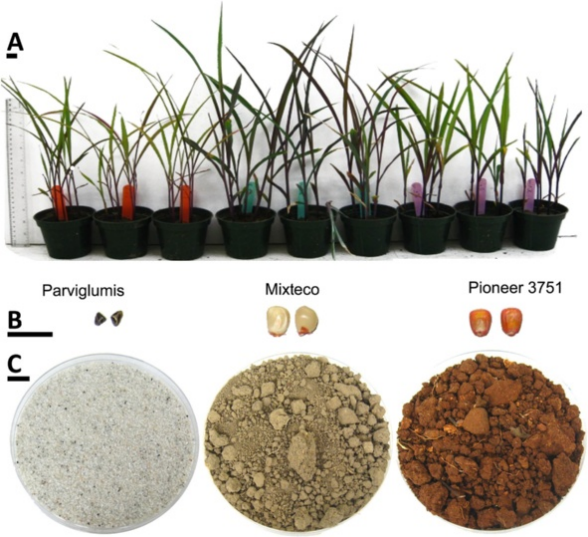
**Seeds, plants, and substrates used in this study. (A)** The three juvenile plant genotypes at the five leaf stage growing in Canadian soil are shown (from L to R): ancestral Parviglumis teosinte (red stakes), traditional Mexican landrace Mixteco (blue stakes), and the modern temperate hybrid Pioneer 3751 (purple stakes). **(B)** Examples of seed are shown (from L to R): Parviglumis, Mixteco and Pioneer 3751. **(C)** Pot substrates are shown (from L to R): sterilized sand, Canadian agricultural soil, and Mexican soil from a Parviglumis field. The scale bars on the left equal 10 mm. For a physical and chemical comparison of Canadian and Mexican soil, see Additional file 1: Table S1.

The objective of this study was to characterize the effects of migration on the founder bacterial endophyte communities of *Z. mays* under controlled conditions. We acquired seed of the three genetically diverse *Z. mays* genotypes described above and grew them side-by-side on three soils: a Mexican, non-agricultural soil in which were found growing wild, pre-domesticated Parviglumis; an agricultural soil from a field growing modern hybrid corn in Canada; and sand that had been heat-sterilized to kill potential endophyte colonists (Figure 1). Bacterial endophyte communities were sampled from roots and shoots by DNA extraction with terminal restriction fragment length polymorphism (TRFLP) fingerprinting and by culturing on nutrient agar. The consequences of soil swaps on the endophyte community profiles of these *Z. mays* genotypes were compared using multivariate statistics. The microbial profiles of sibling seeds, in combination with the plants grown on sterile sand, were used to understand the contributions to the microbial community from inheritance (vertical transmission), while the microbial profiles of the associated soils from Mexico and Canada were used to clarify the microbial contributions of these soils.

## Results

### Physical-chemical soil analysis

Canadian soil (grey-brown Luvisol) was excavated from around the roots of 10 *Zea mays* ssp *mays* (modern maize) plants in a long-term maize trial field, while Mexican soil (mixed Regosol/ Leptosol) was sampled from around the roots of wild Parviglumis plants (ancestral maize) in an uncultivated field in Mexico (see Methods). Soil analysis showed that the Canadian sample was a silt loam soil while the Mexican sample was a clay loam soil, both with a similar pH (pH 7.7 versus 7.5, respectively); the control substrate was sand with a pH of 8.6. The wild Mexican soil had nearly two-fold more organic matter content than the agricultural Canadian soil (3.76%, 2.28%, respectively) and likewise contained higher levels of arsenic, aluminum, cadmium, calcium, iron, lead, molybdenum, vanadium and zinc. The only mineral that was more abundant in the Canadian soil than Mexican soil was extractable phosphorus (Additional file 1: Table S1).

### Bacterial 16S TRFLP profiles of juvenile plants are very different from those of the soil in which they were grown but are somewhat similar to those of seeds

**Figure 2:**
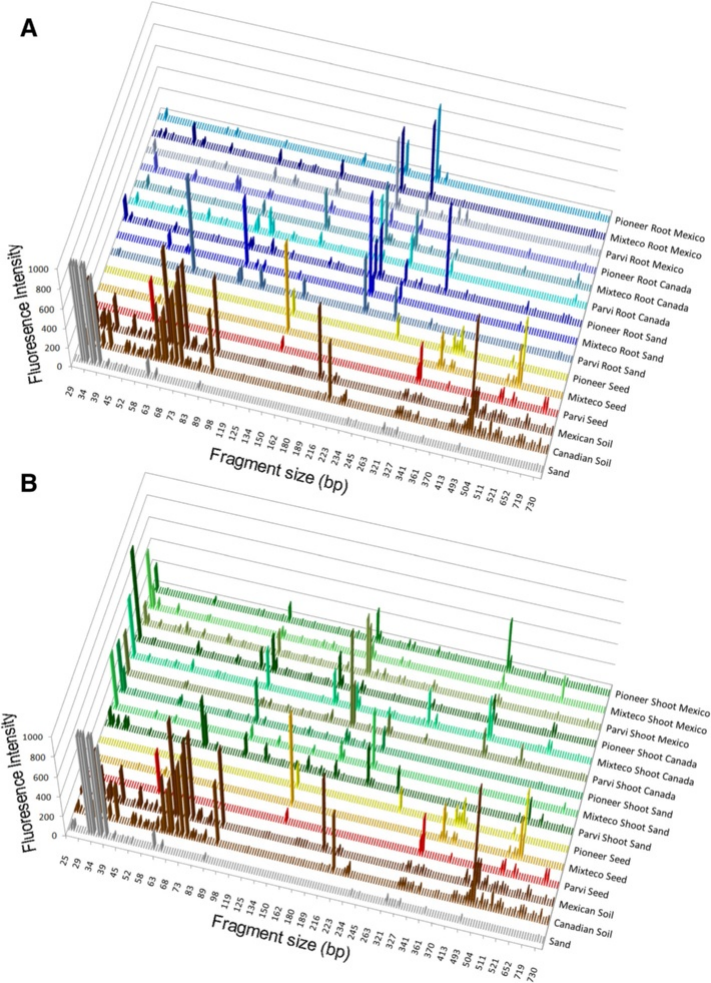
**16S rDNA TRFLP profiles of the bacterial endophytic communities inhabiting young** ***Zea*** **plants based on a culture-independent approach.** Shown are fluorescently labelled (6FAM) 799f fragments of bacterial DNA from: **(A)** shoot tissues and **(B)** root tissues growing in different soils. Each peak is the fluorescence intensity average of six TRFLP amplifications from three pools of five plants, a semi-quantitative indicator of microbial titre. Mixteco plants grown in sand are the average of four TRFLP amplifications from two pools of 5 plants. 16S rDNA amplicons were generated using primers 799f/1492rh and then were restricted using *Dde*I. Small fragments and those corresponding to 16S chloroplast rDNA or 18S rDNA were removed from the display. Max550 labelled fragments are not shown.

The three diverse *Zea mays* genotypes (pre-domesticated/wild: Parviglumis; ancient landrace: Mixteco; modern temperate hybrid: Pioneer 3751) were grown in a growth chamber side-by-side in pots containing either non-sterile Mexican or Canadian soil or heat sterilized sand (Figure 1). At 20 days after germination, roots and shoots were harvested and weighed (Additional file 2: Figure S1). Bacterial DNA fingerprinting of soil and surface-sterilized roots, shoots and seeds was conducted by TRFLP (Figure 2; Additional file 3: Figure S2). TRFLP data was further matched to sequenced 16S rDNA amplicons from cultured bacteria to assist in assigning taxonomic identities (Additional file 4: Table S2). A total of 105 of these sequences (≥200 bp) were submitted to Genbank (accession numbers JF776463-JF776567). Principal component analysis (PCA) of covariance was performed on TRFLP profiles to attempt to explain the causes of any shifts in the bacterial communities (6FAM and Max550 labelled; fragment size presence or absence in 6 PCR trials - Additional file 3: Figure S2).

There were obvious differences in the raw TRFLP fragment profiles observed in roots (Figure 2A) and shoots (Figure 2B) when compared to TRFLP signals from soils and seeds. Soil profiles were dominated by small sized fragments of between 50 to 100 bp in size. As it has been previously shown that soil that is directly attached to plant roots (rhizosphere soil) can be enriched in plant-associated bacterial populations distinct from more distant bulk soil, it is regretful that we did not include this sample type in our study. Seeds had a few large peaks sized from 300 to 500 bp; root profiles were defined by peaks of between 200 to 300 bp in size (Figure 2A); while some of the more striking peaks in shoots were between 100 to 200 bp in size (Figure 2B).

**Figure 3:**
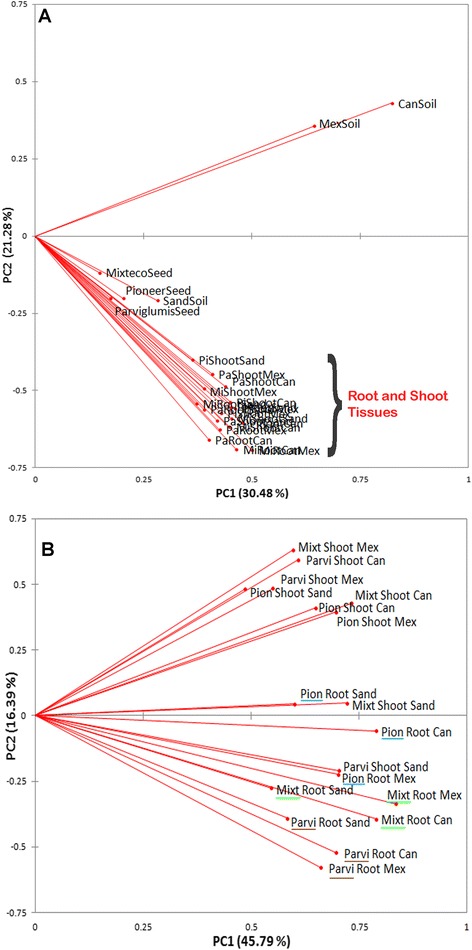
**Clustering relationships between endophytic microbial communities based on principal component analysis (PCA) of bacterial DNA fingerprints (both 6FAM and Max550 labelled 16S rDNA TRFLP fragments).** Shown are endophytic community groupings including: **(A)** soil, seed, shoot and root data; **(B)** only shoot and root data. Parviglumis root samples are underlined in brown, Mixteco roots in green, and Pioneer roots in blue. Results are displayed as biplots of the first and second principal components, with vectors representing the different samples; vector length represents the amount of variation in that sample, and angles between vectors represent the degree of variance between samples. Abbreviations: PI, Parviglumis; MI, Mixteco; PA, Pioneer 3751; Can, Canadian soil; Mex, Mexican soil; sand, sterilized sand.

Multivariate principal component analysis (PCA) of the 16S rDNA TRFLP peaks showed that detectable bacteria resident in shoots, roots, and to a lesser extent, seeds, clustered together, quite far removed from bacteria residing in the Canadian and Mexican soil samples (Figure 3A). Although some TRFLP peaks were shared between plant and soil microbial profiles (Additional file 5: Figure S3B), soil and plant vectors were angled very far away (~90°) from each other (Figure 3A) suggesting that bacterial communities in soil were very different from communities in roots or shoots or seeds. Contrary to the selective endophyte uptake theory, TRFLP profiles of Parviglumis plants grown in their native Mexican soil did not appear to be very similar to the TRFLP profile of the Mexican soil itself. Similarly, TRFLP profiles from the temperate hybrid Pioneer 3751 did not more closely resemble profiles from the Canadian soil compared to the Mexican soil (Figure 3A). These data showed that the bacterial communities of the soils versus plant tissues were dramatically distinct.

### PCA of bacterial 16S TRFLP profiles distinguishes root versus shoot tissues

The PCA analysis was repeated without soil or seed data, which increased the variation explained by PCA from 52% to 62% (Figure 3B). PCA of only root and shoot TRFLP data showed separate clustering of root microbial communities away from shoot communities (Figure 3B). Consistent with this result, β diversity analysis of TRFLP data using Sørensen’s similarity index (QS) showed that 16S TRFLP peaks were significantly more similar between the same tissue across different host genotypes (roots, QS range = 0.63-0.78; shoots, QS range = 0.70-0.81) than between the different tissues belonging to the same host genotype (QS range = 0.49-0.57) (Mann Whitney p = 0.024).

### The composition of bacterial 16S TRFLP profiles observed in plant tissues is more influenced by plant genotype than by pot substrate

Within tissue-specific groupings, root 16S TRFLP profiles were more clustered into host genotype subgroups, and not the pot substrate subgroups as originally expected (Figure 3B), contrary to the hypothesis that the majority of root endophytes are derived from soil. Consistent with this result, TRFLP peaks from roots grown on sterilized sand (autoclaved twice and tested for sterility based on culturing, data not shown) clustered with those from plants that were grown on soils but only when those plants belonged to the same genotype (Figure 3B). However, soil could be seen to have an effect in the PCA, as Parviglumis and Mixteco roots grown on Canadian and Mexican soil were positioned closer to each other, than they were to the roots of the same genotype grown in autoclaved sand. Pioneer roots grown in all three substrates appeared to be spaced equally far apart from each other. No clustering pattern was observed in the PCA of shoot tissues, which appeared to be more randomly organized (Figure 3B).

**Figure 4:**
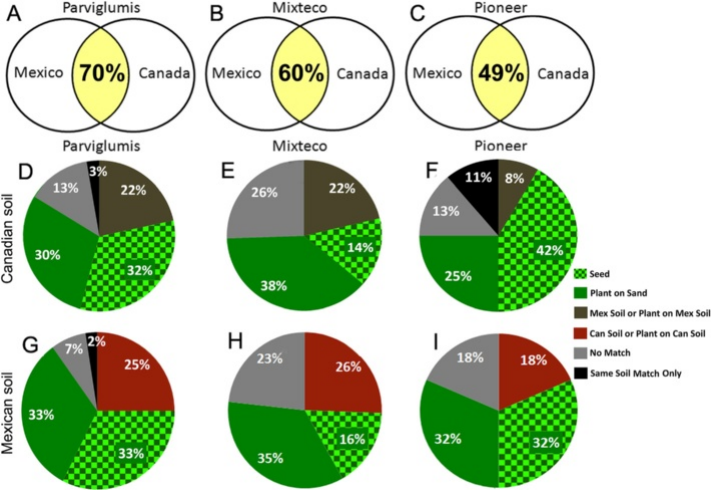
**Relatedness and categorization of TRFLP fragments present in young** ***Zea*** **plants grown on different substrates.** Shown is data from plants grown on Mexican soil versus Canadian soil for: **(A)** Parviglumis, **(B)** Mixteco and **(C)** Pioneer 3751, based on Sørensen’s similarity index of bacterial DNA fingerprints (16S rDNA TRFLP peaks). **(D-I)** Co-occurrence of 6FAM and Max500 labelled TRFLP peaks in different samples: *Seed*: TRFLP peaks in soil-grown plants that are shared with peaks present in seed; *Plant on Sand*: TRFLP peaks in soil-grown plants that are shared with peaks present in sand grown plants but not found in seeds; *Soil or Plant on Soil*: peaks present in soil grown plants, that are shared with the opposite soil and plants grown on the opposite soil, but not in seeds or in plants grown on sand; *Same Soil Match Only*: peaks present in soil grown plants, that are shared with the same soil the plant was grown on, but not in seeds, nor in plants grown on sand nor in plants grown on the opposite soil nor the opposite soil itself; No Match: peaks present in soil grown plants but not found in seeds, sand grown plants, opposite soil grown plants, or either soil. Pie charts D-F show fragment co-occurrence percentages for plants grown on Canadian soil and G-I on Mexican soil.

To quantify the above observations, when the TRFLP peaks (including both 6FAM and Max550 labelled fragments) were compared between plants grown on Mexican soil versus Canadian soil, the Sørensen’s QS value was 0.70 for Parviglumis, 0.60 for Mixteco, and 0.49 for Pioneer, even when combining root and shoot data (Figure 4A-C). Even assuming that multiple microbial species can share the same TRFLP peak size, this high degree of sharing of TRFLP peaks between plants grown on different soils was found to be statistically non-random and highly robust across *Z. mays* genotypes (Additional file 6: Table S3).

When examining root microbial communities separately, Parviglumis roots grown in different soils had a QS value of 0.75, whereas Parviglumis roots versus Mixteco roots grown on the same soil (Mexican) had a QS value of 0.61, while Parviglumis roots versus Pioneer roots grown on Mexican soil had a QS value of only 0.35 (data not shown). At the genus level, the cultured endophyte communities were also fairly similar when whole plants of the same genotype were grown on Canadian versus Mexican soil (QS = 0.58 for Parviglumis; QS = 0.47 for Mixteco, and QS = 0.47 for Pioneer) (data not shown). These numbers support the patterns observed in the PCA, which suggest that host genotype is more important in shaping endophyte communities in young plants than is soil type.

### There appears to be significant vertical transmission of bacteria in both traditional and modern *Z. mays* genotypes

Given the strong effect observed of the host genotype on candidate endophyte populations, the extent of possible endophyte vertical transmission was investigated by analyzing how many TRFLP peaks from soil-grown plants were also present in sand-grown plants and/or in sibling seeds of the original planting materials. An average of 28% of TRFLP peaks present in young plants were shared with their surface sterilized parental seeds (14-42% range) (Figure 4D-I). Sørensen’s similarity index using combined TRFLP data (6FAM and Max550 labelled fragments) from both roots and shoots suggested that the plant endophyte communities were on average ~46% similar between sand and soil grown plants (QS = 0.49, 0.42 and 0.47 for the three *Zea* genotypes). Sørensen’s similarity index also suggested that the cultured bacterial community was on average 47% similar between sand and soil grown plants (QS = 0.46 for Parviglumis, 0.52 for Mixteco, 0.44 for Pioneer). In total, 51-67% of TRFLP peaks present in soil grown plants were present in parental seeds and/or sand-grown plants (Figure 4D-I), suggesting that the largest fraction of bacterial endophytes found in young *Zea* plants were vertically transmitted and not soil derived. With respect to each genotype, the results were similar: a total of 62% and 66% of TRFLP peaks present in soil-grown Parviglumis plants had evidence of vertical transmission, compared to 51% and 52% in Mixteco, and 64% and 67% in Pioneer (Figure 4D-I). The sharing of TRFLP peaks between soil grown plants, and sand grown plants and/or sibling seeds, was found to be statistically non-random, even assuming that different microbial species could result in a common TRFLP peak size (Additional file 6: Table S3).

### *Zea mays* plants appear to be able to uptake bacteria with the same 16S rDNA TRFLP peak sizes from geographically distant soils

Within each genotype, there was an additional class of TRFLP peaks that were shared between plants grown on both Mexican and Canadian soils, which we hypothesized represented the ability of plants to select and uptake taxonomically similar microbes from diverse soils. To characterize this class, TRFLP peaks from soil-grown plants were first filtered out if they were also present in seed or in sand-grown plants (as these represented putative instances of vertical transmission). The remaining TRFLP signals were kept if they co-occurred in plants grown on the opposite soil (or in the opposite soil itself). Based on this classification scheme, for plants grown on Canadian soil, 22% of Parviglumis peaks, 22% of Mixteco peaks, and 8% of Pioneer peaks, were classified as originating from soil and also being shared across geographic locations (Figure 4D-I; statistical analysis in Additional file 6: Table S3). For plants grown on Mexican soil, the shared numbers of TRFLP peaks were: 25% for Parviglumis, 26% for Mixteco, and 18% for the Pioneer hybrid (Figure 4D-I; Additional file 6: Table S3).

### Ancestral, pre-domesticated Parviglumis does not possess more unique bacterial TRFLP peaks when grown on its native Mexican soil than on Canadian soil

Our original hypothesis was that Parviglumis teosinte, the wild ancestor of maize, might prefer to uptake microbes from its native Mexican soil than distant unfamiliar soils, due to co-evolutionary selection. Opposite to this expectation, multivariate analysis showed that Parviglumis, when grown on its native soil, possessed microbial TRFLP profiles that clustered with the profiles of sibling plants grown on Canadian soil (Figure 3). To examine this question more robustly, we individually scored the number of TRFLP peaks in plants that were uniquely associated with growth on its native soil (i.e. not shared with seed or plants grown on sand or Canadian soil); surprisingly, only 2% of TRFLP peaks fell into this class, compared to 3% of unique TRFLP peaks when Canadian soil was substituted (Figure 4D,G). However, amongst the TRFLP peaks observed in soil grown Parviglumis plants, there were additional peaks that could not be explained as either inherited or soil derived (see grey slices, Figure 4D-I). As these may have represented rare soil microbes that were only enriched once they colonized the plant, it was possible they were specific to the soil that the plants were grown on. Even including this potential “error” in the calculation, the results suggested that no more than 9% of candidate endophyte TRFLP peaks observed in Parviglumis were taken up uniquely from its native soil when grown on that soil, compared to 16% when the plants were grown on Canadian soil (grey plus black slices, Figure 4D-I; Additional file 6: Table S3). By comparison, the percentage of soil-derived microbes that might have been unique to a soil ranged from 23-26% for Mixteco (Figure 4E,H) to 18-24% for Pioneer 3751 (Figure 4F, I; Additional file 6: Table S3). Combined, these data do not support the hypothesis that wild, pre-domesticated Parviglumis preferentially takes up microbes from its native Mexican soil.

### A modern maize hybrid does not appear to block entry to soil-derived endophytes

We had hypothesized that modern maize hybrids may have been inadvertently bred to take up fewer microbes from the soil, in order to maintain yield stability across environments. Opposite to this expectation, the TRFLP profiles of the modern Pioneer hybrid were not more clustered across diverse soil environments than the ancestral plant genotypes (Figure 3). To understand this observation, we systematically counted the number of TRFLP peaks in Pioneer 3751 plants that appeared to originate from soil (either unique to a soil sample, or shared by plants grown on both Mexican and Canadian soil). Pioneer 3751 plants contained only a slightly smaller fraction of putative soil-derived TRFLP fragments on Canadian soil (19%) and Mexican soil (18%) compared to either the traditional Mixteco landrace or pre-domesticated Parviglumis plants (range 22-27%) (Figure 4F,I; Additional file 6: Table S3). Given the number of peaks of ambiguous origin (grey slices, Figure 4D-I), we have not found evidence to conclude that a modern maize hybrid substantially rejects more microbes from soil as sources of endophytes than ancestral plant genotypes.

### Culturing predicts the taxonomies of vertically transmitted or soil derived bacteria

**Figure 5:**
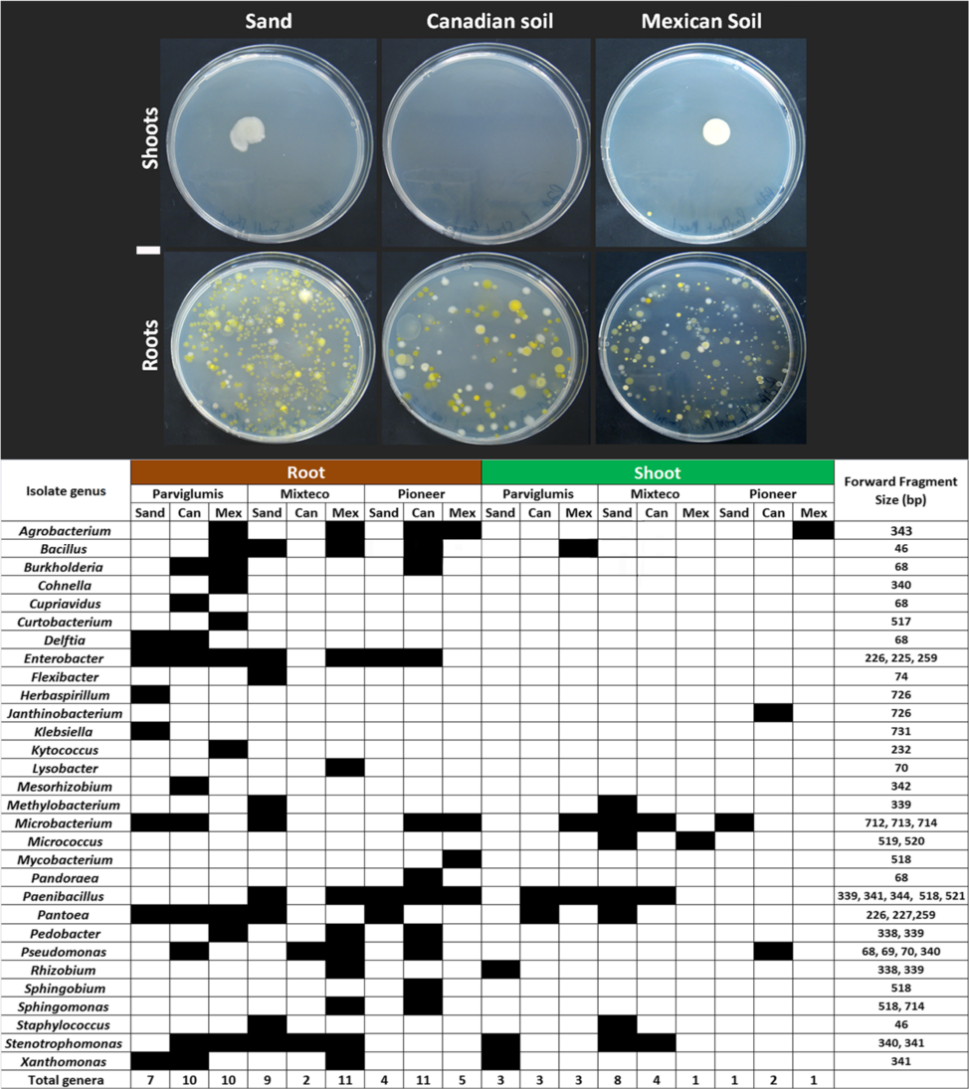
**Summary of bacteria cultured from surface sterilized roots and shoots of** ***Zea*** **plants grown in sand, Canadian soil or Mexican soil.** *Top panel:* Examples of R2A plate cultures of extracts from roots and shoots of Parviglumis when grown on different substrates. *Botton panel:* Taxonomic identification of cultured microbes based on sequencing of 16S rDNA from each colony. A black box indicates successful culturing of that genus. To enable comparisons to TRFLP results, the 16S rDNA sequences were virtually digested and the peak sizes are shown in the right column (predicted 16S rDNA *Dde*I 6FAM labelled cleavage product fragment sizes). The white scale bar on the left equals 10 mm. More details on bacterial isolates are found in Additional file 3: Table S2.

An attempt was made to culture microbes from *Z. mays* samples, in combination with 16S rDNA sequencing, in part to pinpoint the genus-level taxonomies of soil-derived and vertically transmitted microbes. We cultured 124 bacteria from 30 different genera (Figure 5; Additional file 5: Figure S3). The data was not as consistent as TRFLP profiling, but some observations could be made. A subset of microbial genera (*Enterobacter*, *Microbacterium* and *Paenibacillus*, followed by *Pantoea* species, *Stenotrophomonas* and *Bacillus*) appeared to be somewhat conserved across the various host genotype and pot treatment combinations, including plants grown on sterilized sand, suggesting that these microbial genera were inherited rather than soil derived (Figure 5; Additional file 5: Figure S3). Some microbial genera were more associated with a specific genotype: four of the 9 genera (*Enterobacter, Klebsiella, Pantoea,* and *Stenotrophomonas*) cultured on sand-grown Parviglumis (Figure 5) were also only cultured from Parviglumis seed, suggesting these to be vertically transmitted (Additional file 5: Figure S3). Other genera appeared to be soil derived: for example, from Pioneer roots and shoots, *Agrobacterium* species were cultured when the plants had been grown on soil but not sand (Figure 5; Additional file 5: Figure S3).

### The *in vitro* activities of *Z. mays* endophytic communities can be supplied by microbes predicted to be inherited

**Figure 6:**
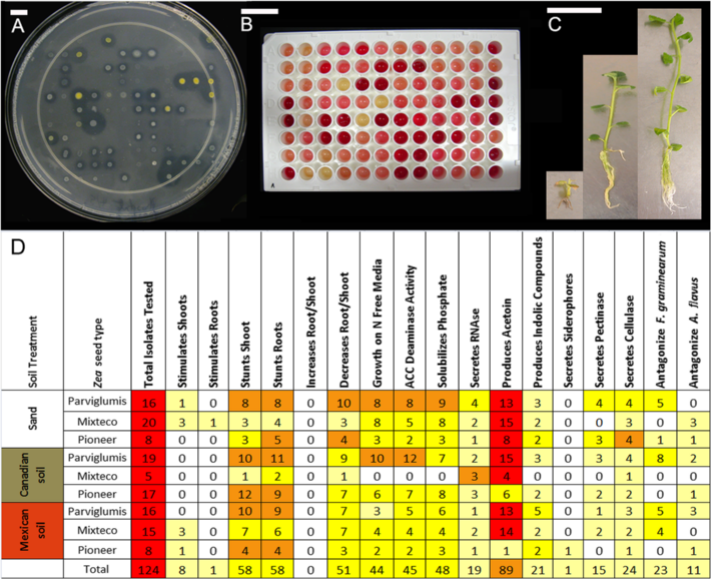
**Analysis of** ***in vitro*** **functional traits of bacterial endophytes cultured from** ***Z. mays*** **plants.** Shown are **(A-C)** select examples of trait assays and **(D)** the complete summary of observed traits organized by host genotype and substrate treatment. Examples of assays are: **(A)** the ability of microbes to solubilise tricalcium phosphate (indicated by a clear halo); **(B)** acetoin and butanediol production (indicated by red colour); **(C)** growth promotion of tissue cultured potato one month after inoculation with (from L-R) *Klebsiella pneumoniae*, sterile buffer or *Methylobacterium oryzae*. For panel **(D)**, isolates were scored as either having activity (1) or not (0) and hence the numbers indicate the number of isolates from that cultured community that express the trait noted. Light yellow shading indicates that <25% of isolates exhibited the trait, deep yellow indicates 25-50%, orange indicates 50-75%, and red indicates 75-100%. The scale bars on the top equal 10 mm.

Plants might not select for endophytic taxa *per se*, but rather the functions of these microbes. Thus, we asked whether the functional traits exhibited by the cultured *Z. mays* endophytic communities, *in vitro*, were affected by the pot treatments. Microbes were characterized for 17 functional traits that could potentially influence host growth and/or health, including: mineral nutrition factors [phosphate solubilisation (Figure 6A), growth on nitrogen-free media as an indicator of biological nitrogen fixation or nitrogen scavenging, siderophore production for iron acquisition]; synthesis of indole-containing compounds which includes the root growth stimulating hormone, auxin [43]; synthesis of ACC deaminase (which catabolizes the precursor of the plant stress hormone ethylene) [44]; and production of acetoin/butanediol [which alters synthesis of the plant hormones, ethylene and cytokinin (Figure 6B)] [45,46]. The ability of *Z. mays* endophytes to promote growth of potato explants that were previously cured of all cultivatable microbes [47] was tested by measuring potato biomass after inoculation with different isolates (Figure 6C). This potato based bioassay was employed since a comparable gnotobiotic tissue culture system had not yet been developed for maize. Tests were also conducted to detect antagonism against potential pathogenic viruses (RNAase secretion) and the maize pathogenic fungi, *Fusarium graminearum* and *Aspergillus flavus.* Finally, pectinase and cellulase activities were tested, since endophytes live in a niche consisting of pectin and cellulose.

The overall results showed that endophytes cultured from soil-grown plants versus sand-grown plant displayed a similar diversity of *in vitro* activities, regardless of whether the host genotype was ancient or the result of modern breeding (Figure 6D). This observation suggests that microbial-derived traits of potential benefit to young *Zea* plants are not dependent on soils as microbial donors but rather can be supplied by vertical transmission. With respect to evidence for co-evolution of *Z. mays* plants with their native soils, there was no obvious difference in the diversity or frequency (when normalized for isolate number) of the *in vitro* traits exhibited by the candidate endophyte communities of young Parviglumis plants that were grown on their native Mexican soil compared to sibling Parviglumis plants grown on temperate Canadian soil or sterilized sand (Figure 6D).

To determine whether host genotype or soil was able to select for different endophytic traits, PCA analysis was also undertaken, similar to the 16S TRFLP analysis. To enable the PCA, the bacterial isolates were assigned binary scores for each trait (positive activity = 1; no activity = 0) (Figure 6D, Additional file 7: Figure S4 and Additional file 4: Table S2). The influence of host tissue type was ignored and shoot/root data were pooled, since very few bacteria were cultured from shoots. The PCA of endophytic activities showed that host genotype had more influence on the types of traits exhibited by members of an endophyte community than soil type (data not shown). For example, 6 out of 8 potato shoot growth promoting endophytes were isolated from Mixteco plants, compared to only one endophyte each from Parviglumis and Pioneer 3751 (in total, 40/124 endophytes were cultured from Mixteco) (Figure 6D). Isolates from Mixteco grown on sand (3/20) or Mexican soil (3/15) showed plant growth promotion (only 5 microbes in total were isolated from Mixteco grown on Canadian soil) (Figure 6D). As Mixteco is a giant plant adapted to grow on low nutrient soils [35], growth promoting bacterial endophytes may play a role in its life strategy.

## Discussion

In an earlier study which examined the endophytes of diverse genotypes of the genus *Zea*, including wild Central American/Mexican teosintes, traditional North American landraces and modern hybrids, we demonstrated that the endophytic communities of seeds remained surprisingly stable after re-growth of plants on a common Canadian field [41]. This result suggested that *Z. mays* can substantially buffer its seed endophytic communities against migration to new soils. In this study, we directly tested this hypothesis by performing soil swap experiments in a controlled greenhouse. We used non-sterile seeds to mimic the activities of plants in the real world. Our most important result, based on multivariate analysis of bacterial 16S rDNA peaks, is that plant genotype rather than pot substrate (sharing a similar pH) plays the most important role in shaping endophyte populations. To understand this result, detailed analysis of TRFLP peak sharing was conducted, which suggested that the proximate sources of candidate founder endophytic bacteria in wild/pre-domesticated plants (Parviglumis), a traditional landrace (Mixteco) and a modern hybrid (Pioneer 3751) were a combination of passive soil uptake, selective soil uptake and vertical transmission. The latter two mechanisms may contribute to the importance of host genotype in shaping endophytic communities, and as a consequence may partially buffer the bacterial endophytic communities of this crop from geographic migration. We did not find evidence to support the hypothesis that wild Parviglumis teosinte preferably takes up microbes from the native soil on which it co-evolved. Parviglumis seeds possess a tough extra layer (fruit case) which we speculate may serve to house and inoculate emerging seedlings with seed-transmitted endophytes [41] that compete against soil-derived microbes. We also did not find evidence to support the hypothesis that the endophytic community of the modern Pioneer hybrid is more buffered than its ancient counterparts against the effects of geographic movement in order to promote yield stability across environments.

### Passive versus selective uptake of endophytes from soils

The Mexican versus Canadian soil swaps had only a modest effect on endophytic communities (Figure 3), and our TRFLP peak sharing analysis suggested that no more than 9-26% of bacterial TRFLP peaks detected in roots and shoots originated from passive uptake of microbes from these soils (Figure 4D-I; Additional file 6: Table S3). By contrast, a surprising conclusion of this study is that young *Z. mays* plants were apparently colonized by similar populations of microbes (based on TRFLP peak sizes) from the two geographically distant soils in a host-genotype dependent manner; 8-26% of the detected 16S TRFLP signals in these plants appeared to derive from both Canadian and Mexican soils (brown and red slices, Figure 4D-I; Additional file 6: Table S3). This promiscuity, at the level of TRFLP, may partially explain why the Mexican versus Canadian soil swaps had only a modest effect on the endophytic communities of these plants (Figure 3, Figure 4A-C).

Soils have been shown to determine bacterial community composition in the rhizosphere [48-50], and traditional thought has assumed that soil would similarly be the major source of endophytes [8]. Indeed, plants have been described as microbial “traps”, able to entice endophytes out from the soils they are growing in [40]. In support of this view, maize landraces grown in soils infected with *Rhizobium etli* from intercropped beans were shown to take up these microbes [51]. In fact, maize root diazotrophs have been shown to be more related to nearby soil communities than to those in shoots of the same plants [52]. Cracks at lateral root branch junctions are the suggested route for systemic endophyte colonization from soil [53] including for the endophytes *Herbaspirillum seropedicae* [54], *Klebsiella pneumoniae* 342 [55] and *Burkholderia phytofirmans* [56]. Environmental entry of microbes has been observed in other plants, including tomato [57].

Our results, however, are more consistent with a recent comprehensive study performed using Arabidopsis [9], which supports the view that the largest fraction of bacterial endophytes is not caused by passive invasion of microbes from soil. In this study, 454 sequencing of 16S rDNA fragments was used to sample endophytes inhabiting 8 *Arabidopsis* genotypes grown from sterilized seed on two types of soil. The results showed that ~60% of the most diagnostic OTUs in the root microbiome were taken up equally from both of the two distinct soils and only the remaining 40% were soil specific; the paper also noted that host genotype played an important role in determining root endophyte communities [9]. This paper supports the idea that there are widespread or promiscuous bacterial endophytes living in soils, but also that there are specialized soil-inhabiting endophytes which different plants are able to selectively take up. It is worth noting that, unlike our current study, the Arabidopsis plants were grown from (very small) seeds which were thoroughly sterilized by treatment with 70% ethanol and bleach before planting [9], which may have biased the study against seed-transmitted microbes.

The observation that geographically different soils can contribute taxonomically similar sources of bacterial endophytes is somewhat surprising given that only 4% of bacteria have been found to be common to geographically distant soils across the Americas [31]. It seems reasonable to speculate that while most bacteria from bulk soil do not have the potential to exist endophytically inside *Zea* plants, soil-derived endophytes originate from a more select subset of microbes which can be found enriched in rhizospheres [58]. Such common endophytes likely include Proteobacteria which account for up to 37% of all maize root endophytes in previous studies [39], and which are commonly observed in diverse soils. It would be interesting to repeat our experiment with soil that has never been associated with *Zea* plants (such as an Arctic gelisol), or soils with diverse acidities, to determine if promiscuous endophytes can still be found.

### Vertical transmission of endophytes

As already noted above, only part of the explanation for why endophytic TRFLP peaks in *Z. mays* plants clustered primarily by plant genotype, and not pot substrate (Figure 3), lay in promiscuous uptake of microbes from soils in a genotype-dependent manner. The major reason for the clustering result appears to be the high fraction of vertically transmitted microbes associated with a specific host genotype: TRFLP peak size sharing analysis suggested that 51-67% of bacterial TRFLP signals observed in young *Zea* plants could be explained by inheritance through seed (Figure 4D-I; Additional file 3: Figure S2 and Additional file 5: Figure S3; Additional file 6: Table S3). Consistent with this result, 17 microbial-derived traits of potential benefit to young *Z. mays* plants were observed from microbes isolated from plants grown on sterilized sand, and hence plants could potentially obtain these traits without depending on soils as donors (Figure 6).

For microbes to be transmitted vertically, they must inhabit seeds, an association which we have recently shown to be strongly influenced by genotype in maize [41]. Similarly, in rice grown on radiation sterilized soil, seed-associated endophytes have been shown to become the dominant endophytic microbes in mature plants, with up to 45% becoming established in the next generation of seed [27]. Interestingly, by using DGGE analysis, this same study showed that soil pH had a major effect on root and shoot endophyte populations. Though soil pH strongly impacts the abundance and diversity of soil bacteria [59], it may have also altered the physiology of potential host plants (e.g. soil pH alters mineral uptake), leading to the selection of different members of the seed derived microbiota. It is noteworthy that both soil types in the rice study were sterile and thus not a major source of observed microbes [27].

Benefits of vertically transmitted endophytic microbes have been observed in seeds of giant cardon cactus, where seed bacteria colonize both the developing seedling and spermosphere where they solubilise rock nutrients crucial for plant growth [23]. In tobacco seeds, microbes were shown to alleviate heavy metal stress in maturing plants [60]. As already noted, to mimic nature, *Zea* seeds in this study were not surface sterilized before being planted, allowing for bacteria living on and underneath the surface of the seed to persist and potentially colonize the germinating seedling prior to soil inhabiting microbes. Early bacterial colonists of eukaryotic hosts have been shown to exert a “barrier effect” against invading microbes [61-63] which may help explain why soil was not as important a source of endophytes as originally assumed. Indeed, ancient versus modern wheat varieties grown on the same soils were shown to have distinct bacterial communities [64]. While founding communities of microbes may help displace late colonizers of the endosphere, the plant itself must also presumably regulate which microbes infect its tissues, or otherwise be overwhelmed by parasites and pathogens.

### The importance of *Enterobacter* and *Pantoea* spp. to the endophytic microbiome of *Z. mays*

Of 70 different, reproducible TRFLP fragments (6FAM labelled) observed at the whole plant level, 9 were conserved across all 9 host genotype and pot treatment combinations, including growth on sterilized sand (Additional file 3: Figure S2) suggesting that these peaks represent seed transmitted bacteria. To predict the identities of these microbes, they were compared to the cultured microbe collection where the 16S rDNA gene was sequenced (Figure 5; Additional file 4: Table S2). The only cultured isolates which matched conserved 16S TRFLP fragments belonged to the genera *Enterobacter* and *Pantoea,* with predicted fragment sizes of 225, 226, 258, and 259 bp; these strains were also cultured from all root genotypes sampled. Consistent with these results, in a previous study of *Zea* seeds, 98% of the cloned sequences with these fragment lengths also belonged to *Pantoea* and *Enterobacter* [41]. Given these independent pieces of data, it is reasonable to hypothesize that these microbial genera are tightly associated with *Z. mays* across diverse environments.

### Study limitations and future experiments

A number of caveats about the methodology used in this study must be noted. First, though we used sequencing of 16S rDNA amplicons to predict the taxonomic identities of microbes cultured in this study, these results did not coincide with all of the TRFLP signals observed, nor can one assume that a TRFLP signal represents a single microbial species, because different bacteria can produce the same 16S rDNA restriction polymorphism. However, as each of our tissue samples typically gave only 35–50 TRFLP peaks from an empirically determined pool size of 314 peaks, the sharing of so many 16S restriction polymorphisms from different tissue samples could not have occurred by random chance, which we demonstrated statistically (Additional file 6: Table S3). Indeed, sharing by different microbial species of a single TRFLP peak size does not increase the probability that TRFLP peaks will co-occur in different tissue samples by random chance, since such contributions also linearly increase the size of the detectable microbiome. TRFLP analysis of microbial communities, in combination with multivariate statistical methods, has been shown to be a robust tool for visualizing microbial differences between samples [65-67] and has been used successfully in a number of microbial ecology studies [41,68-70]. We used TRFLP here as an inexpensive method to analyze the many treatments and replicates required for this study. Nevertheless, it is reasonable to expect that shared TRFLP peaks in plants grown on geographically distant soils might represent, minimally, different strains of the same microbial species or related species. To be cautious, we have tried to refer to our results as “TRFLP peaks” or “16S signals”, and placed predicted species identifiers in brackets. It is important for the reader to understand the conclusions from our study only in the context of the taxonomic methodology employed. Ideally, our study should be replicated using deep sequencing methods along with analysis of DNA polymorphisms at multiple genes.

There were other technical limitations to this study. First, DNA extraction can be variably effective with respect to different bacterial groups, and primer bias in PCR can further skew the view of the community, with studies suggesting that at best, any particular primer pair might only amplify up to 50% of the bacterial diversity in a given sample [71]. Furthermore, it is estimated that any particular microbial strain must be present at ≥1% of the population for molecular fingerprinting to detect it [72], which is why we were careful to note that the study focus was only on detectable endophytes. To encourage amplification of all bacterial groups, here nested PCR was performed twice, totaling 70 cycles, each on three biological replicates (6 replicates per plant genotype-soil combination), and the results were pooled to add weight to 16S signals that were reproducibly higher than background noise; fragments that were observed only once were discarded. To increase fingerprint robustness by including rare groups that were poorly amplified by PCR, we analyzed fragment fluorescence intensity information with a low signal threshold and converted results into binary data to weigh each group equally as recommended elsewhere [73].

Another caveat of this study, as with many endophyte studies, is that we cannot exclude the possibility that we also sampled phyllosphere or rhizosphere microbes. Prior to DNA isolation or culturing, plant tissues were washed and surface sterilized, and the effectiveness of the protocol was verified by culturing the last surface wash on R2A agar plates; no microbes were cultured from plant surfaces. We did not test whether this protocol was effective at destroying bacterial DNA which might have remained on the plant surface; however our protocol (see Methods) exceeded the minimum concentrations and times found to be sufficient for decontaminating bone surfaces of DNA [74] and for cleaning DNA extraction tools [75]. Furthermore, our major result which showed that the Mexican-Canadian soil swap had only a minor effect on the endophytic communities (Figure 3), suggests that our samples had low levels of environmental contamination.

Another technical limitation of this study was that the culture-based analysis of *Z. mays* endophytes may have suffered from under-representation of specific taxonomic groups of microbes. It is well established that many microbes are non-culturable using conventional methods [76]. However, culture based analysis of endophytes was important to include in this study, as it appeared to target a different subset of microbes than DNA based analysis, with only 9 of 73 of the 6FAM labelled TRFLP peaks matching 16 of the 30 genera of cultured microbes (Additional file 3: Figure S2). Also important, bacterial culturing allowed assessment of functional traits from endophyte communities (Figure 6).

The final major limitation of this study is that we analyzed endophyte communities in relatively young plants (~20 days old after germination) and only in the first generation after swapping soils. It is possible that the endophyte communities of young plants are more influenced by founder microbes that are inherited, whereas the titers of soil-derived microbes may increase over time [5,48] until they are perhaps ultimately able to infect future generations through seed. In an earlier study, however, we demonstrated that the bacterial endophyte composition of seeds from nine *Zea* genotypes, imported into Canada from other nations, remained relatively stable when the plants were re-grown on Canadian soil for a full generation [41]. Nevertheless, it would be interesting to characterize how endophyte community composition changes over time when plants are grown on different soil types.

## Conclusions

By germinating pre-domesticated, traditional and modern genotypes of *Z. mays* on geographically distinct soils and sterile sand, we have obtained data that the bacterial endophyte communities of these plants are partially buffered against the effects of geographic migration. Within the limits of 16S-TRFLP profiling, this study suggests that young *Z. mays* plants are not primarily empty receptacles waiting to be colonized by soil microbes, but rather they appear to transmit a majority of their bacterial endophytes from generation to generation through their seed, and further regulate the entry and/or establishment of select and widespread soil bacteria.

## Methods

### Sources of seed

*Zea mays* spp. *parviglumis* (#11355) and *Zea mays* ssp. *mays* var. Mixteco (#24143) were obtained from the International Maize and Wheat Improvement Center (CIMMYT) (Texcoco, Mexico) with the bank accession numbers noted in brackets. CIMMYT seeds were treated with Semevin 350 (insecticide), Tecto 60 (nematocide), and Apron XL (fungicide). Pioneer 3751 seed (Pioneer Hi-Bred T3SZZA11015.00) was kindly provided by Pioneer from a nursery in Szarvas, Hungary. Seeds were treated with Maxim XL (fungicide) and Apron XL (fungicide)*.*

### Sources of soil

Sterile Sand: Nepheline syenite sand (cas# 37244-96-5, Unimin Canada Ltd, Blue Mountain, Canada) was sterilized by autoclaving twice at 121°C for 1 h.

Canadian Soil: A temperate, agricultural silt loam Luvisol was excavated from the rhizospheres of 10 maize plants in a long term maize rotation experiment [77] in Elora, Ontario, Canada, at GPS coordinates 43.641044, −80.405674 on November 4, 2009.

Mexican soil: A tropical, non-agricultural clay loam, Regosol associated with Leptosol, was excavated from the rhizospheres of 10 Parviglumis plants growing in a wild field near Mazatlan, Guerrero, Mexico, at GPS coordinates 17.435517,-99.474068 on October 23, 2009. This soil is from the Balsas River basin and is part of the last wild habitat of Parviglumis [78].

The physico-chemical properties of the two soils were analyzed at University of Guelph Lab Services (Additional file 1: Table S1).

### Plant experimental design and growth conditions

To promote germination (especially important for teosinte), seeds were heat treated in a dry incubator at 40°C for one week, then soaked in distilled water for 24 h at room temperature. For each replicate, five seeds of each genotype were planted in 10 cm wide × 10 cm deep pots containing either soil or sand. There were 3 replicates per genotype, per soil treatment (Figure 1) with the exception of Mixteco on sand which was only replicated twice due to limited seed. Pots were placed randomly in the growth chamber and watered every 24 h with 50 ml of distilled, autoclaved water. Plants were grown to the 5-leaf tip stage (20 days) in a growth chamber with 50% humidity, 16 h photoperiod (200 μmol m^−2^ sec^−1^ at pot height with incandescent and fluorescent lights), with 28°C day and 23°C night. To ensure that the different substrate treatments were not significantly affecting plant growth, fresh tissue weights were recorded at harvest.

### Plant and seed surface sterilization

For root and shoot endophytes, whole plants at the 5-leaf stage (about 20 days old) were carefully shaken free from soil, cut at the root/shoot boundary, washed clean of soil under tap water, placed into separate 500 ml Erlenmeyer flasks (one for the entire shoot and one for the entire root system) and washed in 0.1% Triton X-100 detergent for 10 min with shaking. The water was drained, and samples then washed with 3% sodium hypochlorite for 10 min. The bleach was drained, and washed again in 3% sodium hypochlorite for an additional 10 min. The samples were then drained and rinsed with autoclaved, distilled water, then washed in 95% ethanol for 10 min. The ethanol was removed, and samples rinsed three times with autoclaved, distilled water. To check for surface sterility, one piece of tissue per treatment was transiently placed on sterile R2A agar plates which were incubated for 10 days at 25°C.

For seed endophytes, pools of 15 seeds per genotype were soaked in distilled water for 48 h before surface sterilization with bleach and ethanol as above. To verify surface sterility, 5 seeds per treatment were transiently placed on sterile R2A agar plates, and the plates were incubated for 10 days at 25°C.

### Plant and seed tissue extracts

Surface sterilized tissues (entire shoot and root systems) were ground in an autoclaved mortar and pestle to which was added 1 ml of 50 mM Na_2_HPO_4_ buffer per gram of fresh root or shoot tissue, or per gram of seed weight (Parviglumis seeds received 2 ml/g). 1 ml of each mixture was added to an Eppendorf tube and frozen for later DNA extraction; for culturing, 50 μl of the mixture was serially diluted three times in 450 μl of 50 mM Na_2_HPO_4_ buffer, resulting in 10X, 100X, and 1000X dilutions. The 100X and 1000X dilutions were spread on R2A media (#17209, Sigma) for culturing of oligotrophic bacteria. Plates were incubated at 25°C for 10 days. For later culturing, 400 μl was mixed with 250 μl of 80% glycerol and frozen at −80°C.

### DNA extraction and Terminal Restriction Fragment Length Polymorphism (TRFLP) from plant tissues and soil

Total DNA was extracted from 1 ml of root, shoot or seed extract using DNeasy Plant Mini Kits (Qiagen, USA), and eluted in water. Total DNA from 250 mg of soil or sand was extracted using Powersoil DNA isolation kits (MoBio, USA), and DNA was quantified using a Nanodrop (Thermo Scientific, USA). A PCR mastermix was made with the following components per 25 μl volume: 2.5 μl Standard Taq Buffer (New England Biolabs), 0.5 μl of 25 mM dNTP mix, 0.5 μl of 10 mM 27 F-Degen primer with sequence AGRRTTYGATYMTGGYTYAG [79] (where R = A + G, Y = C + T, M = A + C), 0.5 μl of 10 mM 1492r primer with sequence GGTTACCTTGTTACGACTT [79], 0.25 μl of 50 mM MgCl_2_, 0.25 μl of Standard Taq (New England Biolabs), 50 ng of total DNA, and double distilled water up to 25 μl total. Amplification was for 35 cycles in a PTC200 DNA Thermal Cycler (MJ Scientific, USA) using the following program: 96°C for 3 min, 35X (94°C for 30 sec, 48°C for 30 sec, 72°C for 1:30 min), 72°C for 7 min.

Using the same conditions as above, 1.5 μl of the above PCR product was used as a template in a nested, fluorescently labelled PCR reaction. For the nested PCR, primer 799f with sequence AACMGGATTAGATACCCKG [39] (where M = A + C, K = G + T), was labelled with 6FAM, and 1492rh with sequence HGGHTACCTTGTTACGACTT (where H = A + T + C) was labelled with Max550, both by Integrated DNA Technologies (USA). The forward primer 799 F was chosen as it is strongly biased against amplifying chloroplast 16S rDNA [39]; the much larger mitochondrial 18S fragments were later removed *in silico* after amplification and restriction, but before statistical analysis was performed. 1.5 μl of the labelled PCR product was then added to a 8.5 μl restriction mixture [1U *Dde*I (NEB), 1X Buffer 3 (NEB)] and incubated in darkness at 37°C for 16 h before being analyzed by a sequencing gel using a 3730 DNA Analyzer alongside GeneScan 1200 LIZ Size Standards (Applied Biosystems, USA). There were 3 biological replicates per genotype/treatment combination. For each root or shoot biological replicate, TRFLP amplification and restriction was repeated twice (two technical replicates). Per root or shoot tissue, each genotype/treatment combination thus underwent 6 TRFLP replicates. For seeds, DNA from each pool was subjected to three PCR trials.

### TRFLP analysis

TRFLP results were analyzed using Peak Scanner software (Applied Biosystems, USA) using default settings with a modified fragment peak height cut off of 35 fluorescence units. The forward and reverse fragment sizes plus peak heights were exported to Microsoft Excel. Primer dimer fragments were removed (peaks 1–26 bp).

To generate pseudo TRFLP profiles for display (Figure 2), only forward fragments were used. The TRFLP fragment intensity for each peak was calculated for each PCR trial by subtracting the water control intensity; the results from all PCR trials were then averaged. To generate predictions for the identity of different TRFLP fragment sizes, 16S rDNA sequences from cultured bacterial isolates were submitted to the *in silico* TRFLP analysis program TRiFLe [80]. Additional TRFLP fragment size predictions were generated using the TRFLP analysis program APLAUS + [81].

For PCA, both forward and reverse fragments were used. To reduce experimental noise inherent in TRFLP analysis as recommended by others [73], a fragment size category was only counted if observed in more than one replicate, and the data was formatted as presence/absence counts of PCR trials (0 to 6) in which each peak was detected. PCA of covariance was performed using XLStat software (Addinsoft, France).

Measurements of similarity between microbial communities as indicated by TRFLP were made using the Sørensen’s similarity index (QS), an indicator of Beta diversity which is useful in comparisons between microbial communities [73], using the formula:

$$ \mathbf{Q}\mathbf{S} = \mathbf{2}\mathbf{c}\ /\ \left(\mathbf{S}\mathbf{1} + \mathbf{S}\mathbf{2}\right) $$

where *S1* = total number of species in Community 1, *S2* is total number of species in Community 2, and *c* is the number of species common to the two communities.

### Statistical significance of sharing of TRFLP peaks between samples

To calculate the probability (P) that TRFLP peaks could be shared between different samples by random chance, even when multiple microbial species share the same TRFLP peak size, the following equation was used:

$$ {\mathbf{P}}_{\left(\mathbf{b},\mathbf{c}\right)}={\left(\mathbf{1}/\mathbf{314}\right)}^{\mathbf{c}} \times \left[\mathbf{b}!/\left(\mathbf{b}-\mathbf{c}\right)!\right] \times \mathbf{a} $$

Where:

The fixed integer 314 represents the total number of empirically observed TRFLP peaks in this experiment

a =  number of TRFLP peaks in Sample A
b =  number of TRFLP peaks in the Comparison Sample B
c =  number of TRFLP peaks in Sample A that are shared with Sample B

The derivation for this equation is included (Additional file 6: Table S3), and included in the assumption was that each TRFLP peak size could represent 10 different microbial strains (though the probability is not affected by this variable, since the variable linearly increases the size and hence complexity of the microbiome).

### Taxonomic identification of cultured bacteria using 16S rDNA sequencing

Unique bacteria from each culture plate were chosen based on colony colour and morphology. For taxonomic identification, colony PCR was undertaken as above using primers 27f-Degen and 1492r; when a clean 1465 bp amplicon was present, 1 μl was used directly as template in a sequencing reaction. The sequencing reaction used primer 787f (AATAGATACCCNGGTAG) (where N = A + T + C + G), with an annealing temperature of 49°C, and standard BigDye reaction conditions (Applied Biosystems, USA). If necessary, amplicons were gel purified before sequencing. Reads were BLAST searched against Genbank [82], and 105 of these sequences which were over 200 bp long were submitted to Genbank, and assigned accession numbers [JF776463 - JF776567].

### Phenotyping of cultured bacteria

Agar diffusion was used to screen bacterial endophytes for their ability to inhibit growth of the maize fungal pathogens, *Fusarium graminearum* and *Aspergillus flavus*, *in vitro*. In this method, the endophytes and pathogens were co-plated on Petri dishes: *F. graminearum* or *A. flavus* fungi were first grown overnight (25°C, 100 rpm) in liquid PDA media, then added to melted, cooled PDA media (1 ml of fungus into 100 ml of media), mixed and poured into Petri dishes (100 mm × 15 mm), then allowed to re-solidify. Four holes were created (11 mm diameter) in each plate (in which endophyte liquid solution was later added), by puncturing with sterile glass tubes. Overnight cultures of each bacterial endophyte were centrifuged for 5 min, resuspended in PBS buffer to an OD_600_ of 0.5. For inoculation onto plates, 200 μl of each bacterial suspension was then applied to the holes in the PDA plates that were pre-inoculated with each pathogenic fungus (as described above), then incubated at 30°C for 24 h. The experiment was repeated in triplicate (3 agar plates) and the radius of the zone of inhibition was measured; only endophytes that consistently caused zones of inhibition ≥1.5 mm, were scored as positive. The fungicides Amphotericin B and Nystatin were used as positive controls.

The protocols for the remaining phenotypic tests were identical to those described in an earlier study [41], and used 96 well replica plating when possible. All tests were performed in duplicate, except for the plant growth promotion assay which was carried out in triplicate.

## Additional files

Additional file 1: Table S1. Physical and elemental characteristics of the Canadian and Mexican soils used in this study. Additional file 2: Figure S1. Mean fresh weight of roots and shoots of Parviglumis, Mixteco, and Pioneer plants grown in controlled conditions within a growth chamber either on sterile sand, Canadian soil, or Mexican soil. Error bars show standard deviation. Additional file 3: Figure S2. Summary of 6FAM labelled (799f) TRFLP fragments present in shoots and roots of young maize plants grown in different substrates. Numbers are summed presence/absence counts of 6FAM labelled 16S rDNA TRFLP fragments from six TRFLP replications per sample. Fragment categories were included only if at least one sample had a count of two or more. Potential fragment identities were determined by matching sequenced 16S rDNA amplified from isolates or by APLAUS+. Additional file 4: Table S2. Source tissue, bacterial identities based on 16S rDNA homology, TRFLP fragment sizes, and *in vitro* functional traits of cultured maize root and shoot endophytes. Functions were scored as 0 (no response), 1 (small response), 11 (medium response) and 111 (large response). Additional file 5: Figure S3. Comparison of endophytic bacteria present in *Z. mays* seeds, soils and plants. Both culture-dependent and culture-independent methods are shown. (A) Presence of R2A cultured genus of bacteria in seeds and *Z. mays* plants grown on different substrates. (B) Presence of 6FAM labelled TRFLP fragments in soils, seeds, and plants grown on different soils. Max550 labelled fragments are not shown. Colour shading indicates presence. Additional file 6: Table S3. Statistical probability that TRFLP peaks could be shared between different samples by random chance, when multiple microbial species share the same TRFLP peak size. This table represents the statistical significance underlying the data shown in Figure 4. Additional file 7: Figure S4. Summary of functional traits exhibited by cultured endophytes organized by bacterial genus. Microbes were counted as having activity (1) or being inactive (0) and summed. Light yellow shading indicates that <25% of isolates from the host genotype indicated exhibited the trait, deep yellow indicates 25-50%, orange indicates 50-75%, and red indicates 75-100%.
