# Supplementary figures and images for: Impact of swapping soils on the endophytic bacterial communities of pre-domesticated, ancient and modern maize
Source: BMC Plant Biol. 2014 Sep 12;14:233. doi: 10.1186/s12870-014-0233-3 (PMC4189167; doi:10.1186/s12870-014-0233-3)

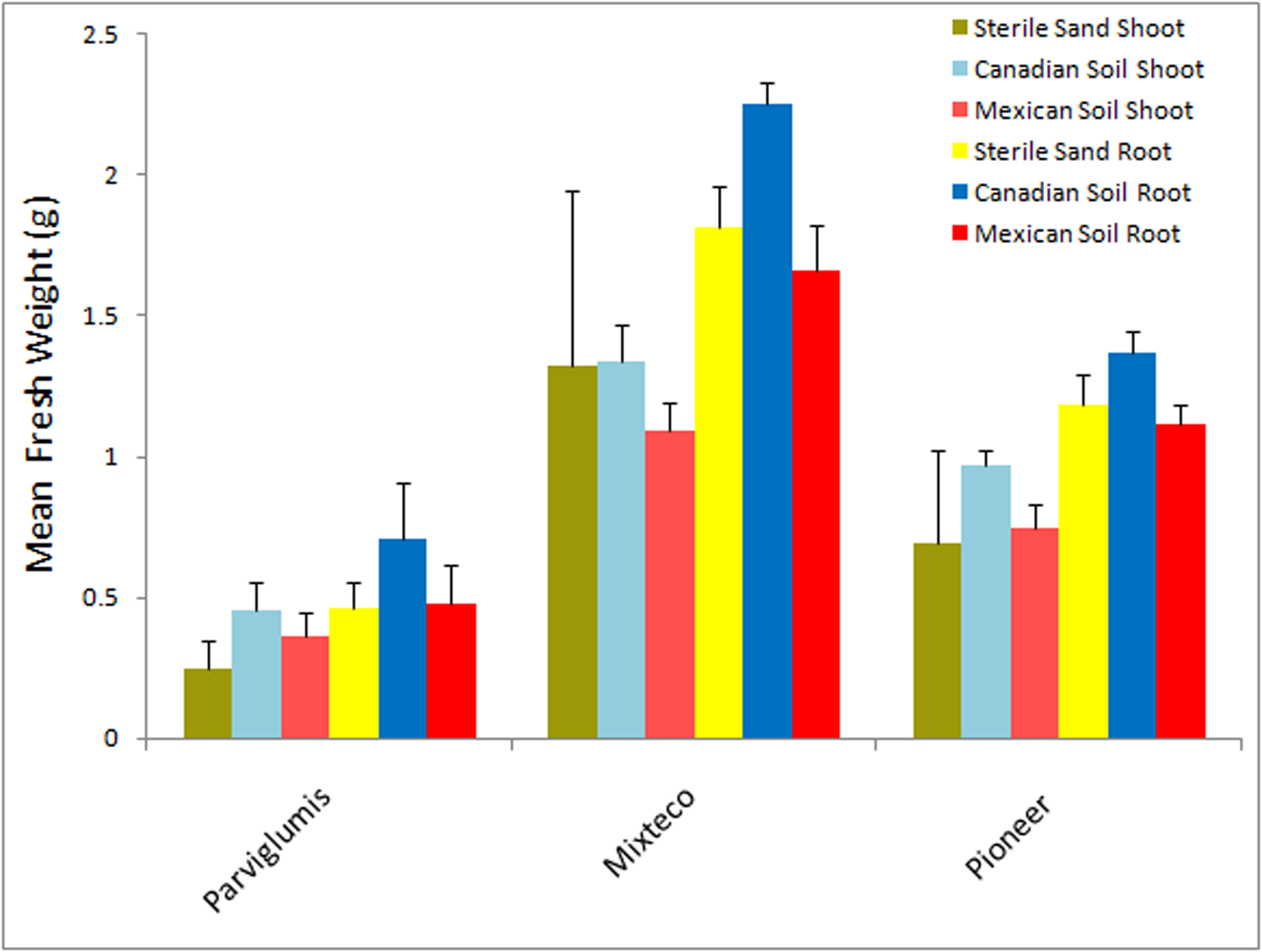

Supplement: Additional file 2: Figure S1. — Mean fresh weight of roots and shoots of Parviglumis, Mixteco, and Pioneer plants grown in controlled conditions within a growth chamber either on sterile sand, Canadian soil, or Mexican soil. Error bars show standard deviation. [file 12870_2014_233_MOESM2_ESM.tiff]

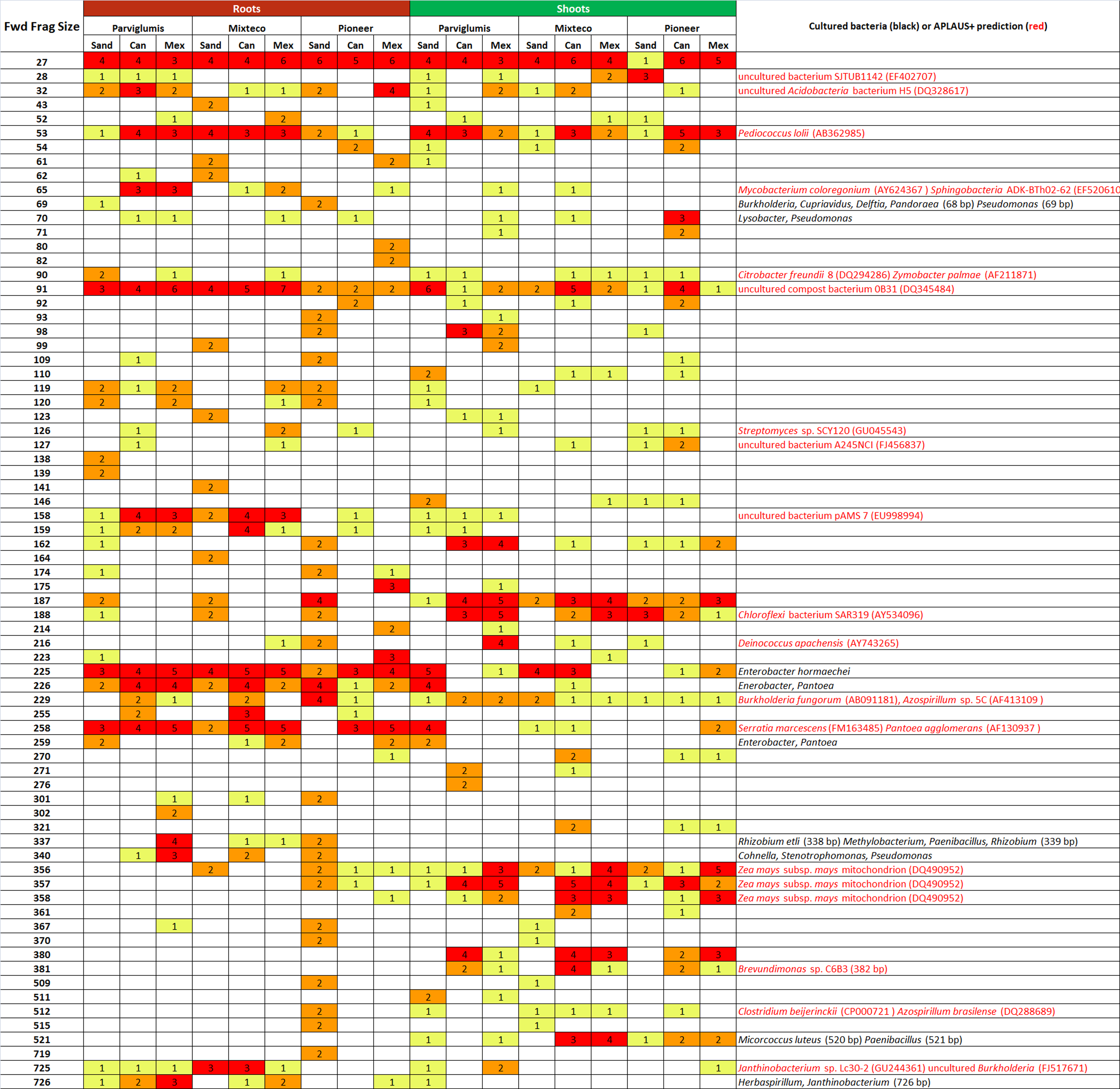

Supplement: Additional file 3: Figure S2. — Summary of 6FAM labelled (799f) TRFLP fragments present in shoots and roots of young maize plants grown in different substrates. Numbers are summed presence/absence counts of 6FAM labelled 16S rDNA TRFLP fragments from six TRFLP replications per sample. Fragment categories were included only if at least one sample had a count of two or more. Potential fragment identities were determined by matching sequenced 16S rDNA amplified from isolates or by APLAUS+. [file 12870_2014_233_MOESM3_ESM.tiff]

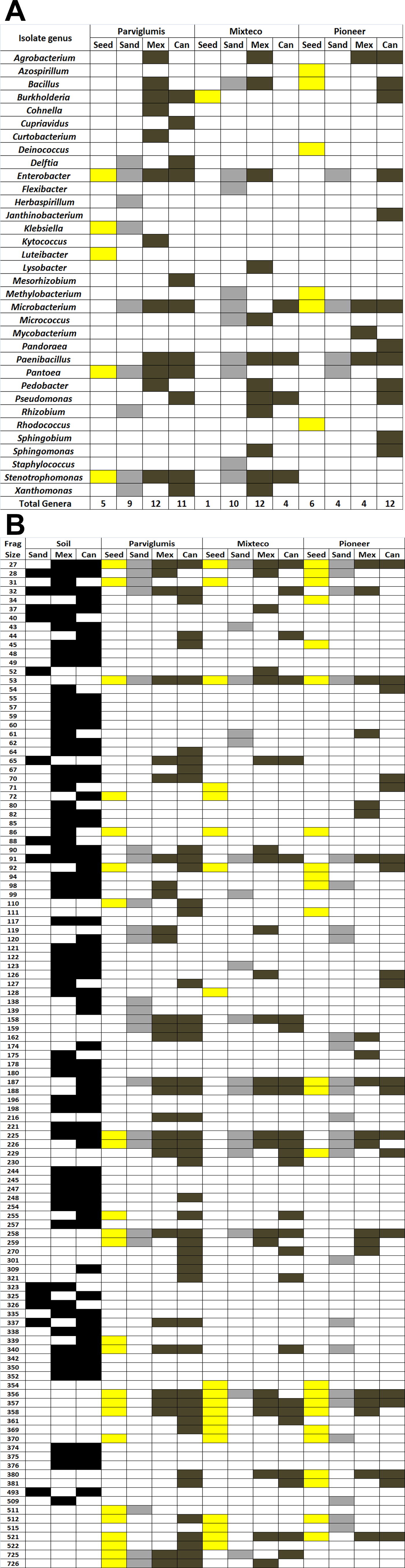

Supplement: Additional file 5: Figure S3. — Comparison of endophytic bacteria present in Z. mays seeds, soils and plants. Both culture-dependent and culture-independent methods are shown. (A) Presence of R2A cultured genus of bacteria in seeds and Z. mays plants grown on different substrates. (B) Presence of 6FAM labelled TRFLP fragments in soils, seeds, and plants grown on different soils. Max550 labelled fragments are not shown. Colour shading indicates presence. [file 12870_2014_233_MOESM5_ESM.tiff]

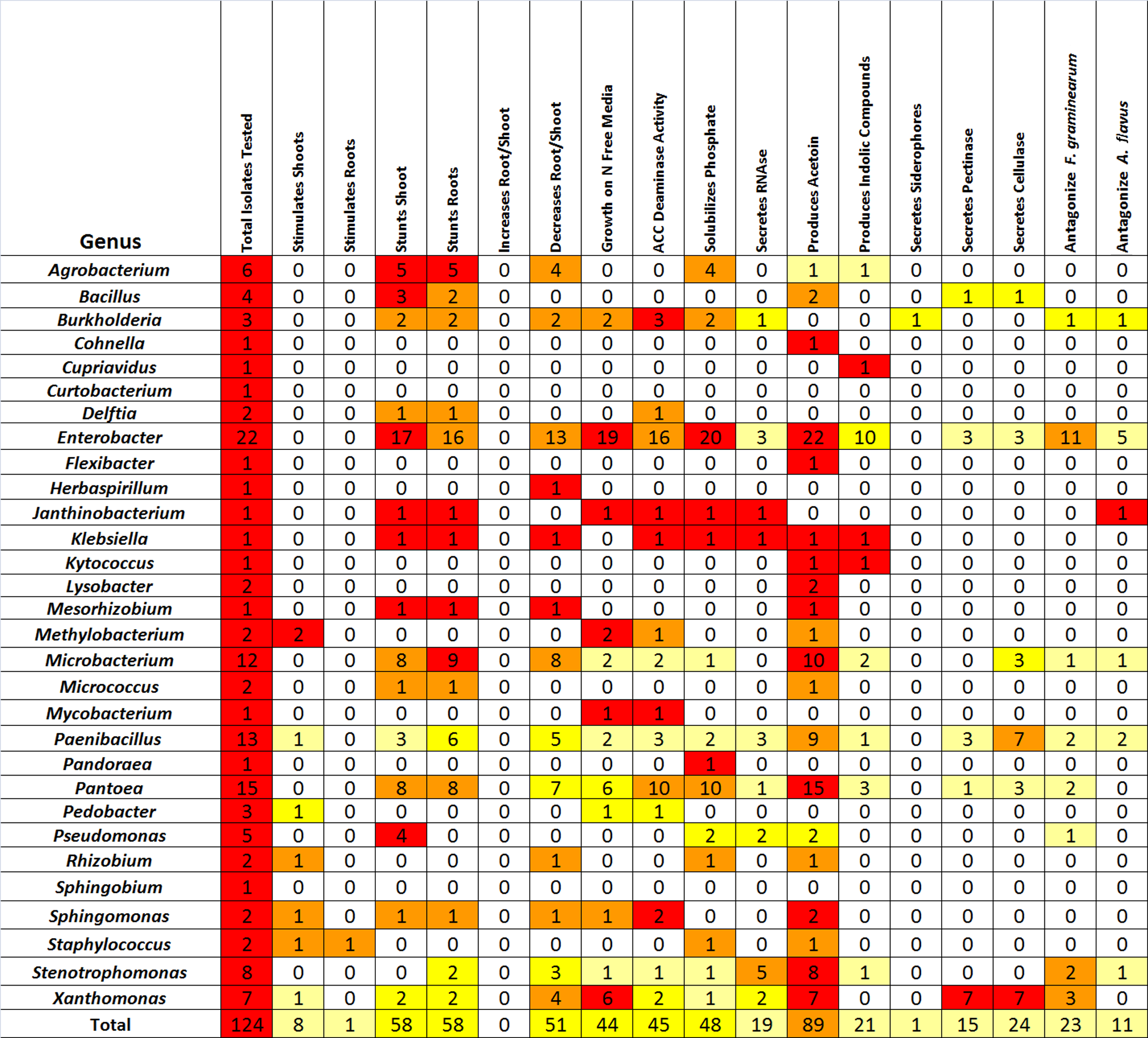

Supplement: Additional file 7: Figure S4. — Summary of functional traits exhibited by cultured endophytes organized by bacterial genus. Microbes were counted as having activity (1) or being inactive (0) and summed. Light yellow shading indicates that <25% of isolates from the host genotype indicated exhibited the trait, deep yellow indicates 25-50%, orange indicates 50-75%, and red indicates 75-100%. [file 12870_2014_233_MOESM7_ESM.tiff]
